# Trees in trees a report from remote Australia

**DOI:** 10.1080/15592324.2023.2286392

**Published:** 2023-12-07

**Authors:** Jane Pye

**Affiliations:** Gingie Station 2422 Gingie Rd, Walgett, Australia

**Keywords:** Artefacts, Billabong, Eucalyptus populnea, murramanaarr paleo-channel, Ngiyampaa nation, songlines, warrambool, woody epiphytes

## Abstract

Anemochory or Ornithochory does not adequately explain the amount, size or distribution of woody epiphytes here in outback NSW Australia. In a semi-arid ecosystem, epiphytes should be short-lived and randomly dispersed not clustered around old Aboriginal campsites or along their ancient paths aka songlines. These enduring trees in trees we call TinTs, have been here much longer than European Australians. We are hoping to attract archaeobotanical or ethnobotanical research to help us recover the knowledge of the ancestors or at least assist us in protecting these arboreal oddities from future resource extraction activities.

I am a farmer from remote Northwest N.S.W, Australia. These are my observations and photographs of what would seem to be an unusually large quantity and variety of “accidental epiphytes” in this area. I call these woody epiphytes ‘Trees in Trees’ or TinTs for brevity and have been uploading their photos taken with my mobile phone since 2019. You can see them in these 2 archives-https://scartrees.com.au/galleries/trees-in-trees/|. https://scartrees.com.au/galleries/cmtints/ I have found over 860 TinTs in the Carinda district where I grew up and west of Walgett where I have lived for the last 36 yrs. I have included this data with exact GPS locations of the TinTs as a supplementary addendum. I am not of Aboriginal descent but have had a long association with the local indigenous community.

The number and combinations of guest and host trees grows every week as I upload the GPS data embedded photos from my mobile phone via my laptop onto google earth. I send the longitude/latitude placements with a brief description to range-land ecologist Dr Jennifer Silcock at the University of Queensland (UQ). Dr Silcock and her partner Russell Fairfax have located about 50 more TinTs in northern NSW and 60 in southern Qld but the vast majority have been found in the area bounded by the today’s townships of Coonamble, Walgett, Lightning ridge, Warren and Brewarrina in far North west NSW. Dr Silcock has submitted a paper to the Australian journal of Botany of which I am the 2^nd^ author. This paper “Unusual, human-mediated prevalence of epiphytes in a semi-arid environment, north-western New South Wales, Australia” is still under review.

I have been contacting Australian universities and museums for the last 8 years and have had no success in attracting any other research. Dr Jonathan Palmer, a Dendrochronologist at BEES (Biological, Earth and Environmental Sciences at the University of NSW), did carbon date a guest slice and a wedge of Cypress pine that was lodged in a remnant Redgum (Eucalyptus camaldulensis – yarraan). There are two relevant and well-respected books but they are both out of print and not available; “Plants of Western New South Wales” by G.M Cunningham, W.E Mulham, P.L. Milthorpe and J.H. Leigh written in 1992 is still the most encompassing source for native vegetation here. The only printed Indigenous cultural knowledge resource I know of for the broader region is “Bush Tucker, Boomerangs & Bandages: Traditional Aboriginal Plant Use in the Border rivers and Gwydir Catchments.” compiled by Michelle McKemey and Harry White.

There is no ancient map of Australian Aboriginal campsites or their songlines (traditional paths). However, some of the oldest roads in this district were originally songlines. White explorers and settlers followed the ancient Aboriginal paths on horseback and horse drawn vehicles. These tracks later became roads for motor vehicles and many were paved/sealed over time. These old roads still have TinTs along their verges and bordering TSRs (traveling stock routes). They often have indigenous names such as Billybingbone rd, Gingie rd and Cumberdoon way.

Indigenous culture & lore was passed down in the oral tradition and there are more than 250 different 'nations' who all spoke different but geographically linked languages. The colonization of Australia resulted in the total annihilation of some Aboriginal “tribes”. I read this recently on a tourist billboard along the Bulgregar creek in the Macquarie marshes – a RAMSAR (International wetland) site – “From 1860 to 1890 the Wayilwan population was reduced from approximately 30,000 to 800 and became almost extinct.” We do know from historical archeological and anecdotal evidence where the large gatherings were held in this area. The Barwon river fish traps at Brewarrina, the Narran lakes (another RAMSAR listed wetland), Cuddie springs and the Macquarie marshes were all visited for ceremony depending on seasonal conditions and the breeding cycles of animals and migratory birds. Unfortunately we don’t have any information about living places at the extended family or clan level pre-colonization. The first Australians were until recently thought to be totally nomadic so unless the landscape contained caves for painting or rocks for engraving no investigations have been conducted.

We do know that Aboriginal people translocated both culturally significant and edible plant species. Dr Jen Silcock, from UQ (the ecologist mentioned previously) has found more than 50 species of trees, shrubs and other vegetation deliberately moved for specific ceremonies and nurtured in their new habitats^1^. Ten of these translocations studied occurred post- contact so Aboriginal people could maintain connection to country if they themselves had been relocated. Many Aboriginal people were removed from their homelands and had historical connections severed. Those claiming ‘first nations’ identity today can often cite heritage ancestries/kin relations with many ‘nations’.

Another cultural tradition I read about recently on my ABC (Australian Broadcasting Corporation) newsfeed concerns the Menang people in Western Australia and their centuries old water – holding trees. According to Lauren Smith (News Reporter – ABC Great Southern) these “Gnaama Boorna” Marrii (Corymbia calophylla) were pruned generations ago to make them hold water for travelling Noongar people. “It’s a practice Aboriginal people have been undertaking for thousands of years, manipulating the trees for their own survival.” Professor Stephen Hopper, University of Western Australia biodiversity expert, called this “skillful horticulture management” and said “it wasn’t just about manipulating trees, but caring for them as well.”

My ethnobotanical knowledge is limited but where possible I will use the common, scientific and Aboriginal names for trees and shrubs when I first mention them and provide a glossary at the end. The three different tribes here often used different names for the same species of animals and plants. We know much about the Ularoi/Youalaroi/Yuwaalaraay people from Katie Langloh-Parker^2^ and the neighboring Gomilaroi/Kamilaroi/Gomeroi were closely related. The Wailwun/Weilwan/Wayilwan across the river were also close kin and part of the Ngiyampaa nation or language group. TinTs are not yet recognized as CMTs (Culturally Modified Trees) under Australian heritage protection laws. There is very little oral history and no written knowledge of this practice. Here is what we know:

**Walgett Elder Allan Tighe** I herd from this area from the old people planted new tree when someone important was born in tribes and in dead parts of tree’s when some person pass on (12 March 2021) Unfortunately Allan has recently had severe health issues and is no longer able to talk or help me look for TinTs.

**Jason Wilson – Youalaroi Gamilaroi Murri (Walgett)** … it was told to me they represent unifying marriage ties to country, in particular Nation boundaries … Youalaroi being dark skin and Gomilaroi being light skin, they are right way marriages … Most Gum trees are Male, the Wilga and Bumble are Female. (15 Feb. 2020)

**Rhonda Ashby (Lightning ridge) and Priscilla Reid- Loynes (Sydney)** As Gamilaroi, Ularoi women we know that trees are Country and family. Trees are one of the many knowledge holders to carry the stories and teachings of our Ancestors. The trees in trees teaches not only about relationships between one another as trees but also about people and Country. As every part of the tree has a purpose not only for its own survival but it has also been a provider for us and our survival as Gamilaroi peoples and all people. Where there are libraries full of books, we have Country with trees full of our cultural knowledge. Trees connects us from the land up into Sky Camp and to all the spaces in between. They were used for birthing, vessels to hold babies and other items, cleansing, seasonal knowledge, medicine and food, portals, shelter, canoes, sign guides, tools and weapons and other uses for animals and plants and of course fire for warmth and heat. (27 March 2023)

The most compelling reason we have for believing the 860+ woody epiphytes/TinTs found here so far are primarily anthropological in origin, is their distribution. Established and enduring TinTs are always associated with other CMTs such as scarred trees, Ringtrees & culturally burnt trees. Australia has the most variable rainfall of any inhabited land on earth. The unreliable precipitation, hot summers and flat topography here means billabongs (waterholes) are often dry. Rivers also fail in prolonged drought and have shifted location over millennia leaving old paleo channels in the landscape. Native wells were dug in the sand along the paleo channel here at Gingie station which was previously known as Moramana from the Gomilaroi word Murramanaarr meaning ‘dragonfly’. The wells were dug to access underground water down to a depth of approximately 9 metres/28 ft. Patches of remnant redgums can be found where the subterranean river is closest to the surface. Redgums are usually only found in NSW along the Murray-Darling basin river system. Most TinTs here are clustered around these Aboriginal wells or in shallow ephemeral swamps and dry sclerophyll forests with billabongs that formed the paleo drainage system for the surrounding plains.

Obligate epiphytes are rare in dry climates with the only true example here being the Black orchid (Cymbidium canaliculatum – garrii). Most gamilaraay, yuwaalaraay, yuwaalayaay tree and plant names are sourced from ‘Bush tucker, Boomerangs & Bandages – Traditional Aboriginal Plant Use in the Border Rivers and Gwydir Catchments’.^3^ Facultative or accidental epiphytes were thought to be restricted to Climbing saltbush – Einadia nutans, Thorny saltbush – Rhagodia spinescens and various grasses, burrs and noxious weeds. However, I have found over 860 woody epiphytes in the last few years with the guests comprising about half the non-eucalypt native scrub species endemic to this area. The host trees here are almost always eucalypts hollowed out by heartwood termites.

There are possible alternative explanations for TinTs not being randomly distributed involving guest species with seeds dispersed by ornithochory. Botanist and expert in arid zone ecology at the University of New England (UNE), Dr Boyd Wright put forward this hypothesis to me recently via e-mail re TinT clusters. “Their profusion around habitation points of the west NSW people could relate to more birds coming into those areas to feed on grass seed that was left behind after people processed grain”. I think the native grass seed of this area was simply too small for birds to see with the commonly harvested grain – Mitchell grass (Astrebla pectinate – ganalay) being smaller than canola seed. The amount of time and effort required to hand harvest and stone grind tiny grass seed would make spillage rare. I’ve seen and videoed flocks of thousands of budgerigars feeding on the Mitchell grass plains here and don’t think they needed human hand outs.

Birds also gather around billabongs to drink but I doubt they would linger due to the extraordinary hunting abilities of Aboriginal people. Even the children had surprisingly developed hand/eye coordination and mastery of the boomerang. Wells dug along the paleo channel were shored up with hollow logs to filter the water and are now the epicenter of TinT clusters. These wells were covered when not used according to Aboriginal Elder Allan Tighe so not a widely available avian water resource.

Not all guest species are bird dispersed, some are wind disseminated. I recently found a solid forked Belah (Casuarina cristata – bilaarr) host with small double guests – Budda (Eremophila mitchellii – badha) and Whitewood (Atalaya hemiglauca – birraa). Budda can be distributed by emus and birds but the winged whitewood seed is spread by wind. There is another Belah host in this swamp as well but the guest is Rosewood (Alectryon oleifolius). I find it unrealistic to attribute these TinTs to natural causes when there are 14 other TinTs in the same shallow swamp and many other traditionally scarred or culturally burnt trees close by.

Another undeniably anthropological TinT is this shallow, normally dry swamp, features an old Emu bush guest, one of the most culturally important medicinal plants found in Australia. (I have only ever found one tentatively labelled Emu bush guest and it could easily be a butterbush, Pittosporum angustifolium – guwiirra/gumbie gumbie, as it was very small and difficult to identify.)

In Aboriginal medical practices, **emu bush** (Eremophila longifolia - ngawil) is highly prized for use in smoking, and scientific research has supported its use as an anti-bacterial, antifungal and antioxidant substance. The leaves of the emu bush are placed on hot embers to produce wet steamy smoke, which kills bacterial or fungal pathogens. This can be of benefit for someone who is sick, to prevent spread of sickness, and for use in childbirth.4

^4^

The long-dead crumbling box tree host has circles engraved around one trunk in regular intervals by a stone axe. There are two shallow irregular-shaped coolamons on the other trunk as well. “A circle or a set of concentric circles usually signify places where people come together. They can represent a meeting place, fireplace, campsite, a waterhole or a ceremonial site.”^5^ Powerful symbols combined with a powerful medicinal plant make this old TinT a significant find. Symbols carved in tree trunks are only transient as the wounds heal over time and are gradually covered with bark. Only termite-resistant species such as eucalypts in dry climates remain standing for many decades. If the tree has died soon after being scarred, the dry face marks are visible until it eventually falls.

For reasons of simplicity and this being a short communication only, I will only discuss the TinTs that I have found here, not those discovered by Jennifer Silcock and her associates. Approximately 90% of the eucalypt hosts are Bimble box/Poplar box – (Eucalyptus populnea – buubaya). The rest are Black box – (Eucalyptus largiflorens – guburruu) and Coolabah – (Eucalyptus coolabah – gulabaa). There is only one Redgum hosted wilga (Geijera parviflora – dhiil) TinT (Figure 1), as well as other rare eucalypt hosts in the bioregions of south west QLD and the central west of NSW.

**Figure 1.**
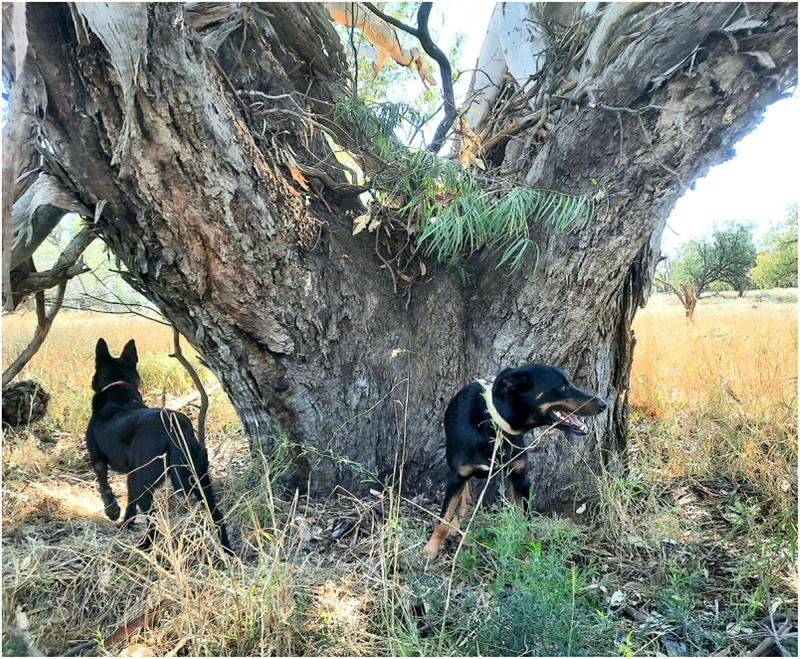
Wilga in redgum on the murramanaarr paleo-channel.

Eucalypts are ageless in the sense that they hollow out over the years so cannot be aged by counting the rings or by carbon dating core samples. Host age is doubly hard to estimate using the assumed semi-arid growth rate of 1–2 mm per year (calculated by Russell Fairfax). Culturally scarred box trees with mature scrub trees growing inside of them obviously have a slower than normal rate of growth. Also many of the bigger eucalypt hosts living on the paleo channels have their roots in the underground water like the redgums so are not retarded by drought. Tree size does not necessarily correspond with tree age along the murramanaarr paleo channel.

Apart from the particular trees, shrubs and ground covers always found around camps there are often stone chips and artifacts as well. There is no natural stone in this area – all is imported. Ringtrees https://scartrees.com.au/galleries/ringtrees/ were also often created at the camps by tying tree limbs together forcing them to inosculate. The branches may have to be retied over generations to achieve the desired shape. These wooden ‘rings’ indicate a reliable water source out here but have different cultural meaning in other parts of the country. Below is a Bumble (Wild orange – Capparis mitchellii – bambul) in Box TinT that is located near an old Aboriginal camp on a neighboring property. Close up you can see how a scar near the base of the host has been cut into and the bumble seed or seedling has been inserted (Figure 2)

**Figure 2.**
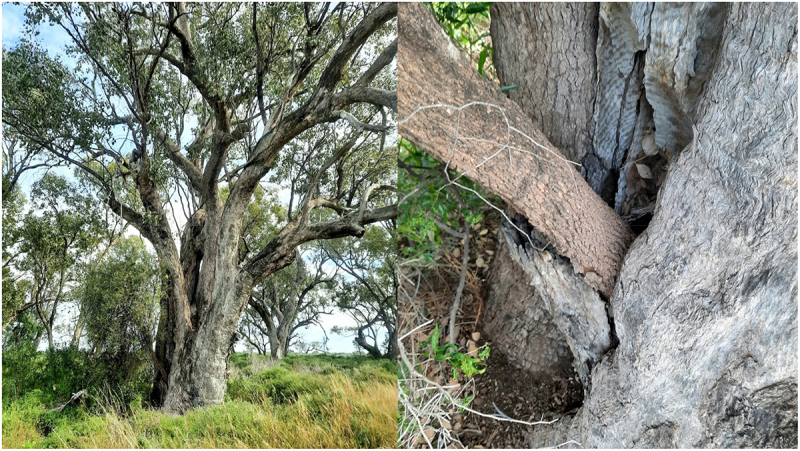
Bumble in box on the murramanaarr paleo-channel.

Dendrochronology also fails when it comes to the age of the TinT guests. Some guests like the rosewood – (Alectryon oleifolius – boonery) fall out of their eucalypt crotches then reshoot from the base. How many times this may have happened before is unknown. I have seen this revival mainly with Rosewood guests in both dead or living eucalypt hosts (Figure 3)

**Figure 3.**
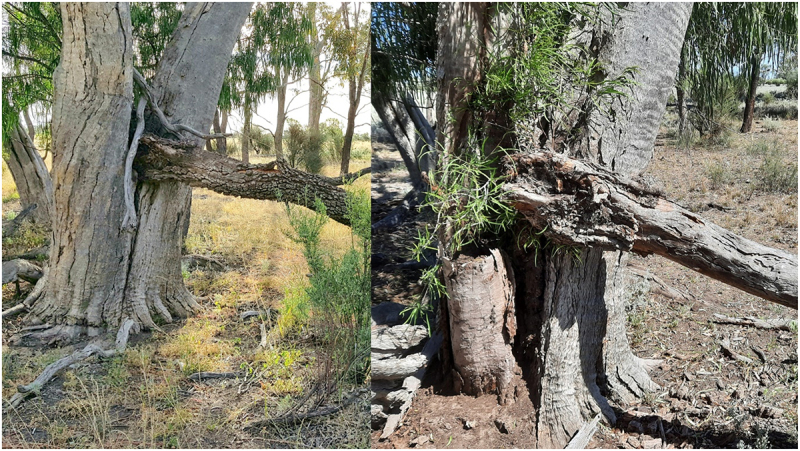
Fallen and reshot rosewood in coolabah swamp.

I have had a 4 cm diameter slice of Quinine – (Alstonia constricta – gadibundhu) guest carbon dated by **DirectAMS (**11822 North Creek Parkway North, Suite 107, Bothell, WA 98,011 USA). The results came back at 40 or 60 yrs old with this caveat^6^

- I will forewarn you that the carbon ratios for the calendar years between ~1600 AD and ~1955 AD are very overlapping. Human activity was putting “dead” or non-radioactive 12C into the atmosphere at an artificial rate. So often the ratios calibrate to 5–7 calendar spans that range from the 1600s to the 50s. If you expect that the trees are around 200 years old or so, that puts you smack in that era. It may be difficult to obtain meaningful calendar calibrations

Below left is the sampled quinine TinT guest where the bimblebox host has had the central trunk removed generations ago. There are two other crotches available on this host, one of which has a tiny wilga – (Geijera parviflora – dhiil) occupant. The photo on the right was taken 2.16 years later showing the quinine reshoot (Figure 4)

**Figure 4.**
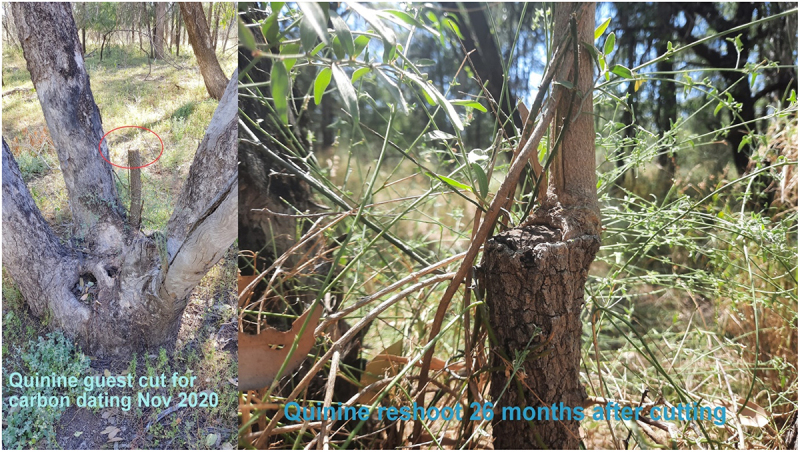
Quinine guest carbon dated by directAMS 2020.

I have recently sent what appeared to be a bone lodged in the scar of a currant/warrior bush – (Apophyllum anomalum – *Capparis anomala* - wayaarra) TinT to direct AMS for ID and carbon dating. Alyssa Tate has replied

As far as we can tell, despite all outward appearances, the entire thing is wood. Both gently scraping off and stabbing an X-acto knife into the “bone-like” surface to obtain a sample for what should be collagen extraction exposes wood. While it is common for minerals to replace the structure of bones (this is how we get true fossils), we are unaware of that occurring with plant growth, so it seems that this tree has just mimicked the look of bone on the exterior of this part

Results from the macrobotanical identification report^7^ just received from Kathryn Puseman (P.A.S.T) are as follows; “This sample did not exhibit any wood anatomical structures typically seen in cross, tangential, or radial views of wood. The fragment did contain structures that appeared somewhat like the thickening ridges seen in the cross sections of epiphytic roots and appeared to have grains of sand embedded in the structure … … It is possible that the wood structure has collapsed to the point it is no longer identifiable or that this fragment does not contain the secondary xylem and phloem structures of typical wood” (Bone mimic extracted from old Currant bush TinT and sent to DirectAMS. Presently awaiting carbon dating results – Figure 5)

**Figure 5.**
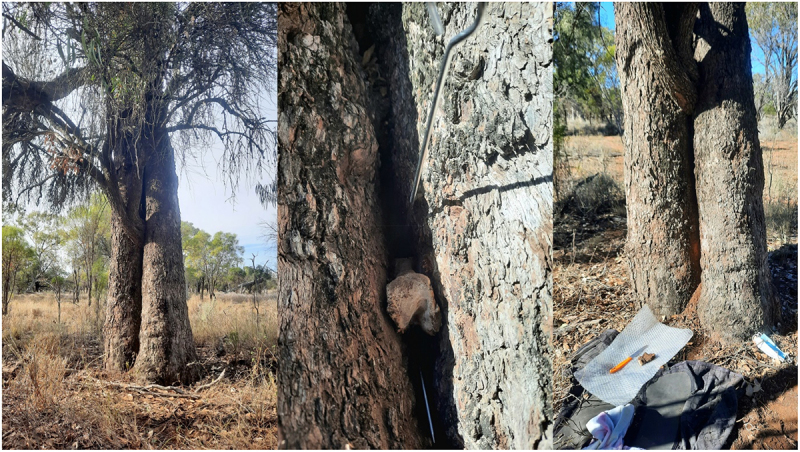
Currant bush TinT with bone like object lodged in the scar.

Conditions inside old eucalypt crotches vary enormously regarding soil quantity and amounts of humus and termite frass present. A very small percentage of guests do not live in hollowed out eucalypts but are found in solid trees. Here are two examples of Wilgas growing in solid Ironwood hosts – (Acacia excelsa – dhan.gayan.gan) (Figure 6).

**Figure 6.**
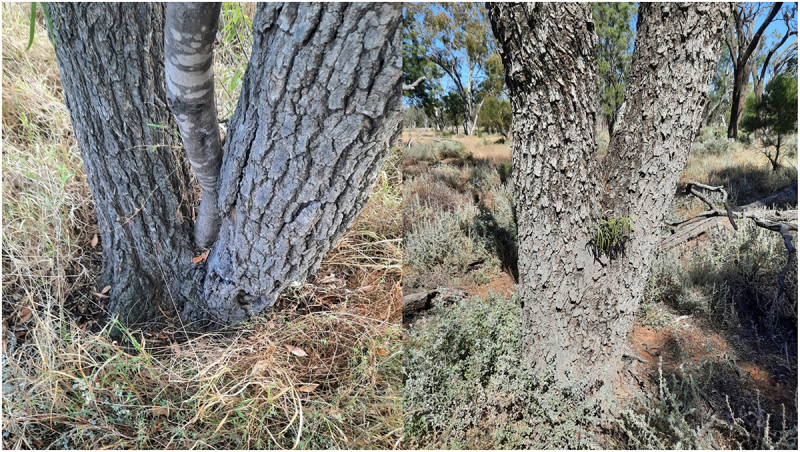
Wilga guests growing in ironwood – solid and hard timber.

Some TinT guests like this old wilga in black box have become root bound and have broken open and killed their hosts (Figure 7).

**Figure 7.**
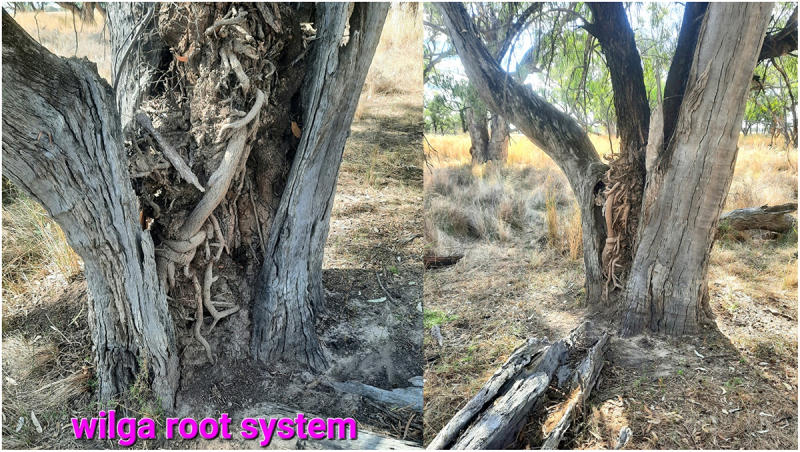
Old wilga in dead black box on the marra ck paleo channel.

Hosts and guests can compete for resources over a very long time as you can see with the wilga – bimble box combination below. The wilga guest has invaded one of the split trunks. There is another of these combinations 30 m away of similar size except the wilga emerges much lower down in a single bimble box trunk (Figure 8).

**Figure 8.**
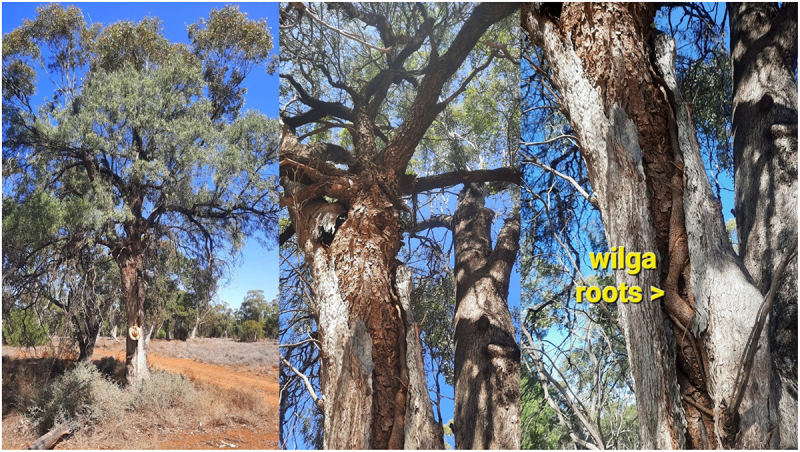
Wilga high up in split box TinT along the cumberdoon way.

Basically, we need to know if it is possible that TinTs such as those featured and on my website could be naturally occurring. The annual rainfall in the TinT regions is between 400 and 450 mls (16”−18”) per year. The mean maximum summer temperature here is 35 Celsius (95 F). We have had less than 150 mls (6”) in the first three quarters of this year and reached 37.4 C (99 F) in mid spring. Science is not supposed to be subjective but as a farmer and gardener I find it hard to imagine TinTs arising spontaneously in wet seasons and surviving the long dry periods without human intervention. Science is meant to be predictable and with the help of Google earth and some local landholder knowledge I can guess with reasonable accuracy where I will find TinT clusters.

Eucalypts themselves are rarely guests and if observed are more likely to be reshoots of the dead host. I have been recording all TinT combinations I find even if the host or guest has died. Occasionally I’ve come across double-deads where the both host and guest are no longer living. Often the host species can be guessed at but unless the dead guest has some remaining bark I have to record it as unknown. Over the last few years, I’ve noticed more guests dying than hosts. From my observations, I think eucalypts live much longer than the common scrub trees such as the wilga. Wilga does not tolerate floods even while living as a guest and some have died along the overfull creeks. High water levels in our regulated rivers are sometimes artificially maintained in La Nina years due to risk of Dam failure upstream.

Google earth screenshot showing 860 TinTs in 28,000 square km – far north west NSW, Australia (Figure 9)

**Figure 9.**
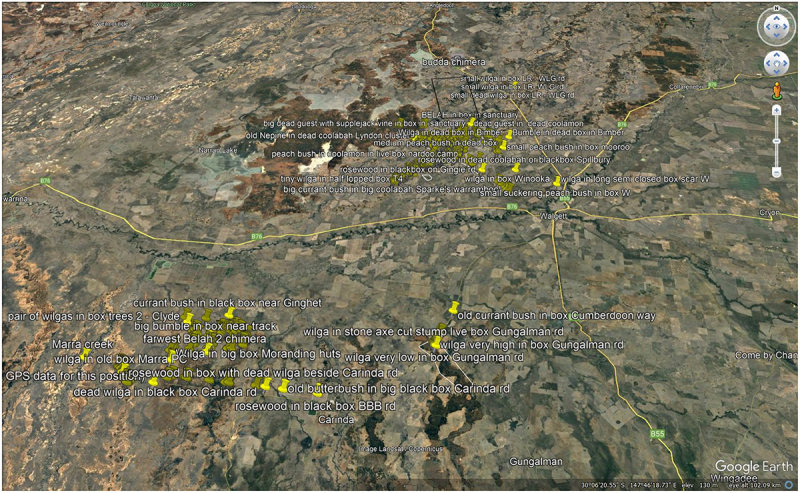
Google earth screenshot showing 860 TinTs in 28,000 sq. km.

A hollow host is obviously a eucalypt and you can predict the species by where it is located. Bimble box grow on the plains and higher woodlands because they don’t like water logging. The black box is more tolerant of inundation and thrives in the swamps and flood plains of North West NSW. Coolabahs also grow in swamps and on heavy clay soils preferring flood plains or riverine environments. Redgums aka River red gums only live on river banks out here or in patches along the murramanaarr paleo channel where the underground water is close enough to the surface. Some TinT combinations are joined at the base but are of similar size so it is difficult to tell which tree is the host and which is the guest. I call these 50:50 trees like the bimble box/redgum (Figure 10).

**Figure 10.**
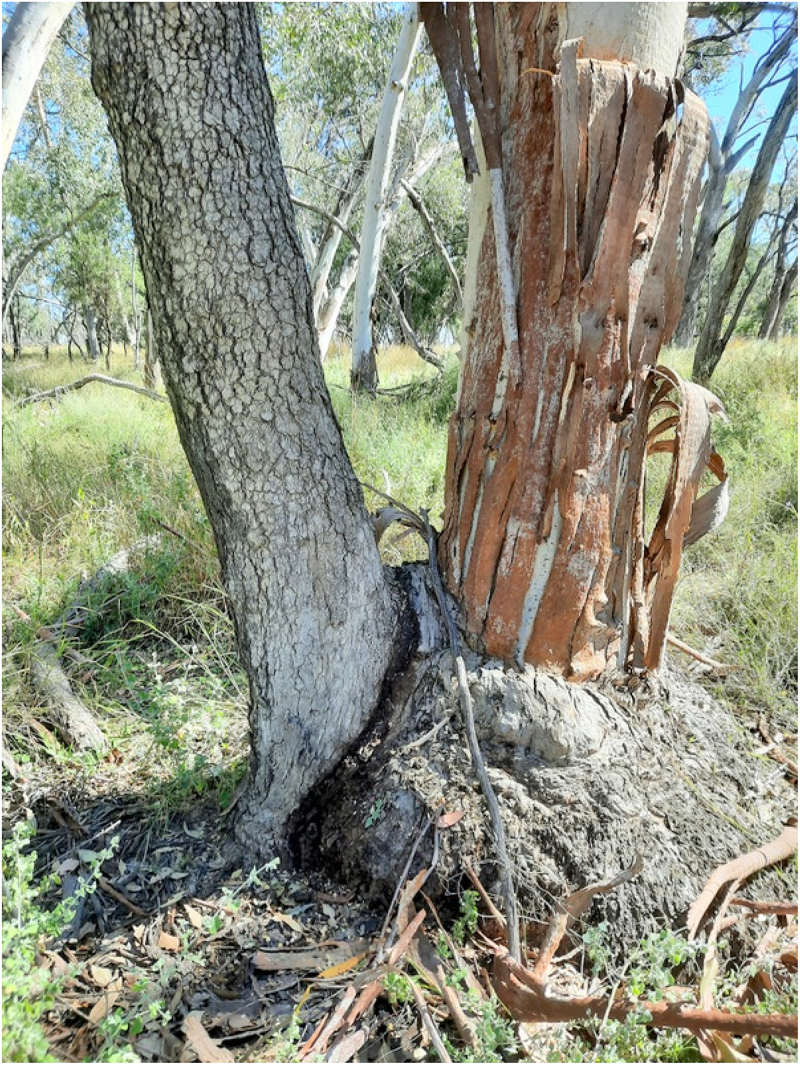
Redgum and box 50:50 TinT – murramanaarr paleo channel.

Some of the TinT hosts here have been poisoned in the 1980s and are collapsing now, like this Butter bush (Pittosporum angustifolium – gumbie gumbie) TinT. The roots of the guest remain exposed in their original position like a stranded asset (Figure 11).

**Figure 11.**
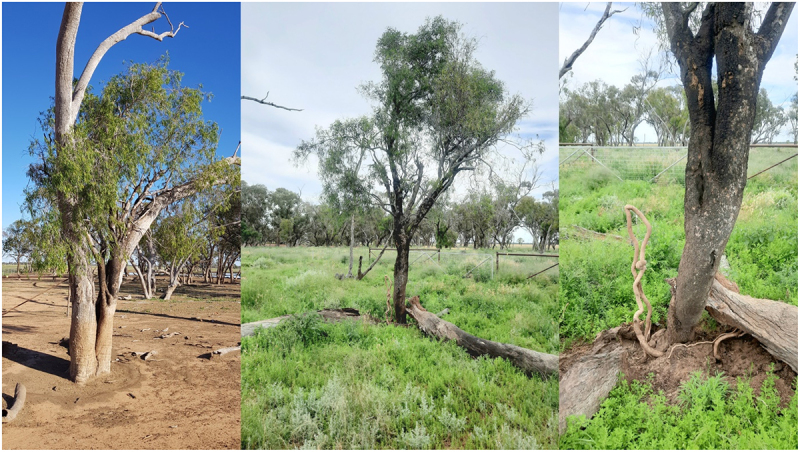
Butterbush in poisoned box near the feedlot billabong.

Of the 860 or so TinTs/woody epiphytes found in this region around 70% of the hosts have been anthropologically modified. Some of these eucalypts have been altered firstly by the local Aboriginal people and then by the European settlers in the last 200 years. Squatters and farmers cleared land and harvested timber whereas the Indigenous people used both the bark and wood for shelter, weapons, tools and cultural needs but rarely killed the trees. Many of these scars on the box trees are still visible but coolabah and redgum heal over relatively quickly so it’s possible nearly all hosts were modified at some stage. We do not need help analyzing scarred trees and other CMTs. We have the oldest continuing culture in the world but some practices like TinT creation have not survived colonization.

Another confirmation of the anthropological nature of these TinTs is the existence of hosts with multiple guests, either in the same crotch or growing in separate locations on the trunk. The largest TinT combining the host and guest circumference would probably be a wilga in box where the host is 4.3 m and the guest 1.15 m around (14 ft and 3 ft 9”). There are larger hosts and larger guests but for this combination to be the result of ‘Mother Nature’ seems unlikely. What are known in the literature as ‘accidental epiphytes’ have never been considered to be deliberate despite a 114-year-old Nothofagus nervosa growing on the canopy of a Chilean rainforest^8^. An Australian study in 2020^9^ looked at accidental epiphytes in a Tasmanian rainforest to assess the effects of moisture availability and host size but not possible anthropological origins.

Vincent Hoeber and Gerhard Zotzcome do suggest “the effect of human landscape management and global change on accidental epiphytes deserves more attention in future research”^10^ This is more in regard to frequent pruning and other horticultural techniques that can lead to a buildup of organic matter in tree crowns so encouraging accidental epiphytes. No one has ever suggested that our ancestors may have grown intentional epiphytes for their own purposes. However, I have read about the 14/15^th^ century Japanese practice of using bonsai techniques on Kitayama cedar. Known as ‘daisugi’ this ancient forestry management technique increases both the quality and quantity of saplings produced from the ‘mother’ tree.^11^

The closest I have come to finding any mention of unusual epiphytes in low rainfall areas was published in the *Queensland Naturalist* in 2014 by Dr Ian Baird^12^.Dr Baird found a Leafless cherry/ballart – (Exocarpus aphyllus – mirrii) growing in an old coolabah branch in 2013. This was found “growing in the most elevated part of the floodway” on Weribone creek in the Maranoa region of QLD. Coincidentally, this is exactly where the local Aboriginal people would have historically camped. Exocarpus aphyllus belongs to the sandalwood (Santalaceae) family and is hemiparasitic on roots. Dr Baird has this to say regarding its possible viability “In this semi-arid environment that would require it to initially persist essentially epiphytically through long dry periods and ultimately for it to have successfully grown roots the length of a hollow in the branch, then down inside a connecting hollow in the trunk of the host and into the ground before attaching to roots in the typical terrestrial hemiparasitic mode. However, such a scenario would presumably require the seedling to have survived well beyond the species’ capacity to survive on its own roots without the resources available from a host”. I’m suggesting the only reason this Exocarpus aphyllus guest survived living in a coolabah branch “well beyond the species’ capability” is that the local people both planted and watered it. Below you can see this particular TinT combination and another example here on the Big warrambool (overflow watercourse) but the coolabah host is dead (Figure 12).

**Figure 12.**
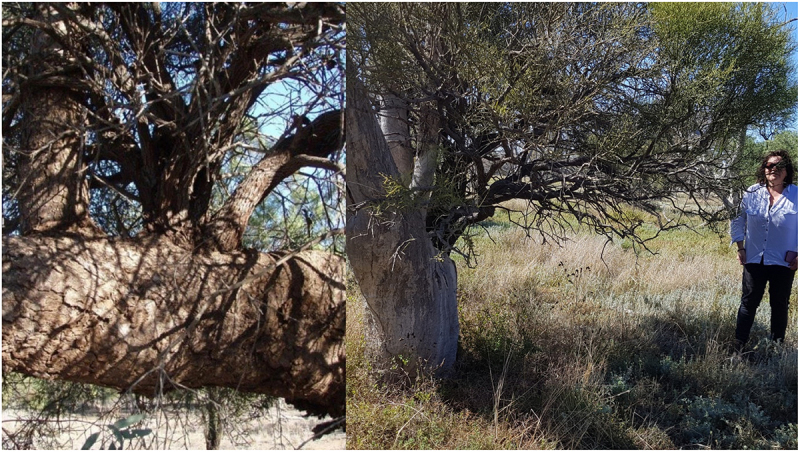
Mirrii TinTs at weribone ck qld and the Big warrambool.

Ive also found Exocarpus aphyllus living in a chest height crotch in a live bimblebox host. Again rather than growing as a root parasite, the guest is living epiphytically. Many of the guest species have delayed germination mechanisms in their seeds. Some require passage through an animal gut or heat or abrasion to sprout. Beth Gott^13^ from Monash University in Melbourne sums this up wonderfully “Aboriginal people have interacted with the Australian flora for many thousands of years. It is well to bear in mind that the evolutionary history of many of the species used by them may reflect that interaction.”

I am not denying the existence of accidental epiphytes in Australia. Even in a 16” (400 ml) average rainfall zone I’ve seen a coastal white cedar (Melia azedarach) growing in an unknown eucalypt that was purchased from a nursery and not naturally occurring in the area. This is located in a garden near a storm water overflow pipe from the roof gutter so it is hardly surviving in natural conditions. There are many examples of accidental urban epiphytes in Australian towns and cities with most involving exotic or invasive foreign species. To my knowledge, none of these opportunistic “guests” mature and persist for generations.

## Discussion

This is what Tex Skuthorpe, Nhunggabarra artist author and educator^14^ had to say about Indigenous land management. ‘Neither the explorers, nor the early settlers who came after them, realised that much of the land and the vegetation they encountered was not natural, but altered by Aboriginal cultivation. The Australian landscape was to a large degree an Aboriginal artefact created by thousands of years of sustaining the earth’. Author and historian, Bill Gammage^15^ reveals an astonishingly intricate and scientific system of Indigenous land management before European settlement. Their awareness of the requirements and life cycles of native plants combined with the mosaic burn regime known as fire stick farming guaranteed plenty of food. We think the Aboriginal people here also used their extensive botanical knowledge to create TinTs for cultural or spiritual reasons and other purposes now best described as permaculture.

As previously mentioned, I am a farmer and citizen scientist not an academic. I would appreciate some help especially in the field of plant signaling chemicals, Allelopathy and mycorrhizal relationships. What interaction if any is there between host and guest? Just 2 out 20 guests account for almost 50% of the more than 860 TinTs here. Is this because the ancestors preferred wilga and peach bush or were they simply the most successful combination. Peach bush suckers readily so some of these TinTs are probably not true epiphytes. Wilga does not sucker and can die if the box tree host is damaged or poisoned. Of the 20 or so native scrub trees in the district that do not grow as epiphytes – were they not wanted or did they fail to thrive? Is it possible to grow citrus and nut trees in old eucalypts? How did a semi-nomadic people in a semi-arid climate nurture the guests? The questions are endless but available time is not. When I leave here there will be no knowledge of or access to the TinTs anymore. The ancient Aboriginal wisdom will be lost for good just when it looks like we might need it to survive this century.

If we could attract a PHD student or researcher I could supply accommodation on our property gratis. Interested members of the local indigenous community, Elders groups and Land councils would be available for interview. This is not cultural appropriation but a plea for help and a leap of faith.

## Supplementary Material

Copy of All TinTs up to Nov 2023.xlsx

## GLOSSARY

**Guest species growing to maturity as epiphytes:**

Boobialla – Myoporum montanum – buubiyala

Budda – Eremophila mitchellii – badha

Butterbush – Pittosporum angustifolium – guwirra/gumbie gumbie

Currant/Warrior bush – Apophyllum anomalum – wayaarra

Cypress pine – Callitris glaucophylla – gurraari

Emu bush – Eremophila longifolia – ngawil (rare)

Leafless cherry/ballart – Exocarpus aphyllus – mirrii

Leopardwood – Flindersia maculate – bagala (rare)

Peach bush – Ehretia saligna – unknown Aboriginal name

Quinine – Alstonia constricta – gadibundhu

Rosewood – Alectryon oleifolius – boonery

Shrubby rice flower – Pimelea microcephala – unknown

Supplejack – Ventilago viminalis – ganayanay

Whitewood – Atalaya hemiglauca – birraa

Wild orange/Bumble – Capparis mitchellii – bambul

Wild lemon – Psydrax oleifolia – unknown Aboriginal name (rare)

Wilga – Geijera parviflora – dhiil

**Guest species rare or not seen growing to maturity:**

Belah – Casuarina cristata – bilaarr (only 1)

Milgee – Acacia oswaldii – boree (rare)

**Climbers:**

Desert/Native jasmine – Jasminum lineare – unknown Aboriginal name

Gargaloo – Parsonia eucalyptophylla – gugumadharraa

Nepine/Wait-awhile – Capparis lasiantha – ngaybaan

**Shrubs: (Aboriginal names are unknown)**

Climbing saltbush – Einadia nutans – unknown (common)

Spiny peppercress – Lepidium aschersonii – unknown (only 1)

Spiny fan flower – Scaevola spinescens – unknown (only 1)

Spiny potato bush – Solanum ferocissimum – unknown (only 1)

Thorny saltbush – Rhagodia spinescens – unknown (common)
